# [Retracted] The oncogenic roles of 27‑hydroxycholesterol in glioblastoma

**DOI:** 10.3892/ol.2024.14829

**Published:** 2024-12-02

**Authors:** Lu Liu, Mei-Yuan Li, Yu Xing, Xiao-Yun Wang, Yong Wang

Oncol Lett 18: 3623–3629, 2019; DOI: 10.3892/ol.2019.10690

Following the publication of this paper, it was drawn to the Editor's attention by a concerned reader that certain of the sphere growth assay data shown in Fig. 4A on p. 3626 had already appeared in a previously published article written by different authors at different research institutes in the journal *Cancer Research*. Upon performing an independent analysis of the data in this paper in the Editorial Office, it was subsequently observed that certain of the data shown in Fig. 2A were also stikingly similar to data that had been published previously in a different article written by different authors at different research institutes. Owing to the fact that the contentious data in the above article had already been published prior to its submission to *Oncology Letters*, the Editor has decided that this paper should be retracted from the Journal. The authors were asked for an explanation to account for these concerns, but the Editorial Office did not receive a reply. The Editor apologizes to the readership for any inconvenience caused.

